# Correlates of Taxane-Induced Neuropathy, an Electronic Health Record Based Observational Study

**DOI:** 10.3390/cancers15030754

**Published:** 2023-01-26

**Authors:** R. Dixon Dorand, Neil S. Zheng, Rajiv Agarwal, Robert J. Carroll, Samuel M. Rubinstein, Karen M. Winkfield, Wei-Qi Wei, Jordan Berlin, Xiao-Ou Shu

**Affiliations:** 1 Division of Hematology and Oncology, Department of Medicine, Vanderbilt-Ingram Cancer Center, Vanderbilt University Medical Center, Nashville, TN 37203, USA; 2 Department of Biomedical Informatics, Vanderbilt University Medical Center, Nashville, TN 37203, USA; 3 Yale School of Medicine, Yale University, New Haven, CT 06510, USA; 4 Division of Hematology, Department of Medicine, Lineberger Comprehensive Cancer Center at University of North Carolina, Chapel Hill, NC 27599, USA; 5 Department of Radiation Oncology, Vanderbilt University Medical Center, Nashville, TN 37203, USA; 6 Department of Medicine, Meharry Medical College, Nashville, TN 37208, USA; 7 Division of Epidemiology, Department of Medicine, Vanderbilt Epidemiology Center, Vanderbilt University School of Medicine, Nashville, TN 37203, USA

**Keywords:** peripheral neuropathy, chemotherapy, taxane

## Abstract

**Simple Summary:**

Chemotherapy-induced peripheral neuropathy (CIPN) is a common adverse effect of taxane chemotherapy. We investigated the associations of demographic and treatment variables with the risk of developing CIPN using electronic health record data. We found higher doses, more taxane cycles, female sex, overweight and obesity, or a history of diabetes were associated with developing CIPN in patients treated with taxane, while concurrent chemotherapy or concurrent radiotherapy were related to a reduced risk. Further studies are needed to uncover the underlying reasons for the observed associations.

**Abstract:**

Background: Chemotherapy-induced peripheral neuropathy (CIPN) is a common therapeutic complication affecting cancer patients’ quality-of-life. We evaluated clinical characteristics, demographics, and lifestyle factors in association with CIPN following taxane treatment. Methods: Data were extracted from the electronic health record of 3387 patients diagnosed with a primary cancer and receiving taxane (i.e., paclitaxel or docetaxel) at Vanderbilt University Medical Center. Neuropathy was assessed via a validated computer algorithm. Univariate and multivariate regression models were applied to evaluate odds ratios (ORs) and 95% confidence intervals (CIs) of CIPN-associated factors. Results: Female sex (OR = 1.28, 95% CI = 1.01–1.62), high body-mass index (BMI) (OR = 1.31, 95% CI = 1.06–1.61 for overweight, and OR = 1.49, 95% CI = 1.21–1.83 for obesity), diabetes (OR = 1.66, 95% CI = 1.34–2.06), high mean taxane dose (OR = 1.05, 95% CI = 1.03–1.08 per 10 mg/m^2^), and more treatment cycles (1.12, 95% CI = 1.10–1.14) were positively associated with CIPN. Concurrent chemotherapy (OR = 0.74, 95% CI = 0.58–0.94) and concurrent radiotherapy (OR = 0.77, 95% CI = 0.59–1.00) were inversely associated with CIPN. Obesity and diabetes both had a stronger association with docetaxel CIPN compared to paclitaxel, although interaction was only significant for diabetes and taxane (*p* = 0.019). Increased BMI was associated with CIPN only among non-diabetic patients (OR:1.34 for overweight and 1.68 for obesity), while diabetes increased CIPN risk across all BMI strata (ORs were 2.65, 2.41, and 2.15 for normal weight, overweight, and obese, respectively) compared to normal-weight non-diabetic patients (*p* for interaction = 0.039). Conclusions: Female sex, obesity, and diabetes are significantly associated with taxine-induced CIPN. Further research is needed to identify clinical and pharmacologic strategies to prevent and mitigate CIPN in at-risk patient populations.

## 1. Introduction

Chemotherapy-induced peripheral neuropathy (CIPN) is a common side effect of cytotoxic chemotherapies used for treating multiple cancer types including breast, gastrointestinal, genitourinary, head and neck, and lung cancers. CIPN is commonly observed among patients treated with platinum, taxane, or vinca alkaloid-based regimens. Due to the widespread use of taxanes across cancer subtypes, taxanes were reported to account for approximately 24–27% of all chemotherapy-treated grade 2 or worse neuropathies [1,2]. Symptoms of CIPN include pain, numbness, tingling, cold sensitivity, and sensory or motor dysfunction, frequently resulting in dose reductions or treatment delays [2,3]. Therefore, CIPN may affect the quality-of-life for patients actively receiving cancer treatment and may also have long-term consequences during cancer surveillance and for survivorship. While many patients experience CIPN, it is often underreported by patients, and symptoms can develop weeks to months after completion of therapy [2,4]. Furthermore, despite recognizing and medically managing symptoms, the oncology workforce often inadequately assesses and underreports low-grade CIPN [4,5]. By understanding the risk factors that lead to the development of CIPN, clinical guidelines can be developed to identify patients susceptible to neuropathy before, during, and after treatment, and help guide treatment protocols to mitigate the risk. 

CIPN onset and severity is often a cumulative effect of cytotoxic chemotherapy, correlated with increased therapeutic dose, duration of treatment, and combination chemotherapy [6,7]. Other factors implicated in developing CIPN include sex, age, body mass index (BMI), race, anemia, hypoalbuminemia, and diabetes [2,8,9,10,11,12,13]. In particular, patients over the age of 65, females, Black race, and obesity (often defined as BMI > 25) were also reported to be associated with both taxane-induced and platinum-induced CIPN. 

As advances in both targeted and immune therapy continue to improve survival outcomes in patients with cancer, the negative impacts of long-term treatment side effects have emerged at the forefront of clinical research. One emerging area of research is the relationship between diabetes mellitus (DM) and CIPN. Over 25% of patients with DM will develop diabetic sensorimotor polyneuropathy (DPN), a condition which clinically mimics CIPN and is also associated with increased BMI [14,15]. Therefore, providers may avoid CIPN-inducing regimens in patients with DPN to preserve their quality-of-life. Recent studies focused on the correlation of comorbidities or independent risk factors for developing CIPN have provided conflicting results when evaluating DM as a risk factor for taxane-induced CIPN. After adjusting for concurrent treatment modalities, a cohort study of 2420 patients with breast cancer showed that DM was not associated with patient-reported taxane-induced CIPN [16]. These results contrast with a meta-analysis including eight trials that revealed an increased incidence of taxane-induced CIPN (OR 1.47 (95% CI 1.11–1.93) in patients with DM albeit demographics, comorbidities, or treatment specific variables were not adjusted in all studies included in the latter [13]. 

The discrepancies among recent studies investigating risk factors for CIPN highlight the clinical need to delineate patient characteristics to inform systemic treatment decisions. Therefore, we conducted an electronic health record (EHR)-based study to comprehensively evaluate both demographic and clinical factors in the context of detailed treatment regimens to identify risk associations with CIPN. 

## 2. Methods

### 2.1. Study Population

Our study included 3387 patients of any age who were diagnosed with a primary cancer and received a taxane (i.e., paclitaxel or docetaxel) at any point in their cancer therapy as part of a treatment plan from Vanderbilt University Medical Center (VUMC). Patients were identified through the Synthetic Derivative, a data repository containing deidentified EHRs for over three million individuals, with records dating back to January 1993 [17]. This study was approved as a human subject except investigation by the Institutional Review Board at VUMC. 

### 2.2. Study Variables and Covariate Assessment

The study variables and covariate assessment were defined in a previous study with the same study population [18]. Briefly, taxane-treatment information was extracted from medication administration records, including overall taxane-treatment start date, mean dosage, mean treatment duration (months), and number of treatment cycles. We focused on only the first taxane treatment course for each individual. Cancer type was determined from the most recent diagnosis code prior to cancer treatment and grouped by anatomical site. Cancer-stage data were retrieved from the VUMC North American Association Central Cancer Registry.

Demographic information was retrieved from EHRs and included: self-reported race, age at first taxane treatment, sex, most recent body mass index (BMI, weight in kilograms/height in centimeter^2^) measurement prior to first taxane treatment, ever smoking status, and history of diabetes. Age at first taxane treatment was categorized into <55, 55 to <65, 65 to <75, or ≥75. BMI was categorized into underweight (BMI < 18.5), healthy weight (BMI ≥ 18.5 to < 25), overweight (BMI ≥ 25 to <30), or obese (BMI > 30). Due to the small sample size for underweight patients (*n* = 102), they were excluded from the current study. Additional covariates retrieved from the EHRs included prior and concurrent chemotherapy (Table S1). Prior chemotherapy was defined as any chemotherapy regimen prior to the first taxane treatment period. Concurrent chemotherapy was defined as any chemotherapy regimen during the first taxane treatment period prior to the first incidence of CIPN.

### 2.3. Study Endpoint

The primary study endpoint was any occurrence of CIPN during and up to 6 months after completion of an individual’s first taxane treatment course. We restricted the study period to six months to minimize the influence of loss to follow-up. We defined CIPN as any grade 2 or 3 CIPN, which is defined by the U.S. National Cancer Institute’s Common Terminology Criteria for Adverse Events, v5.0 as “moderate to severe symptoms that limit instrumental activities of daily living”. CIPN is typically documented in the free text of clinical notes but differentiation between mild CIPN vs. grade 2 or 3 is often not clearly indicated. We trained an L1-regularized logistic regression model to predict CIPN status using a subset of 242 individuals with a manual review of CIPN assessment by a clinician. Variables used to train the model included diagnosis codes, key terms extracted from clinical notes with natural language processing, and changes in taxane dosage, which may indicate dosage adjustments for patients that experience CIPN. We evaluated the area under the receiver operating characteristic curve (AUROC) with five-fold cross-validation. The five-fold cross-validation of the neuropathy status prediction model had a mean AUROC of 0.861 (range: 0.715 to 0.962). Additional details on the model can be found in the Appendix A. CIPN can take months to resolve, and patients may not report symptoms until after the end of treatment regimens [4,19]. Therefore, we applied the CIPN prediction model to all 3387 patients with data from the start of taxane treatment up to six months after the end of taxane treatment.

### 2.4. Statistical Analysis

Pearson’s χ^2^ tests for categorical variables and Student’s *t* tests for continuous variables were performed to compare characteristics of patients who received taxane treatment by incidence of CIPN. Logistic regression models with different adjustments were applied to evaluate odds ratios (ORs) and 95% confidence intervals (CIs) of CIPN associated with categorical age, sex, self-reported race, categorical BMI, history of diabetes, ever smoking status, co-chemotherapy, and co-radiotherapy. All categorical variables were treated as dummy variables in the analysis. Additional multivariate analyses were performed to evaluate associations of CIPN with categorical BMI and history of diabetes with further stratification by taxane drug type (paclitaxel vs. docetaxel) and sex (male vs. female) to evaluate effect modification and joint effects of these variables. Lastly, we evaluated the joint effect and interaction between categorical BMI and history of diabetes on CIPN. *p*-value for interactions was derived from calculating the *p*-value of the F-test comparing the reduced model without interaction term and the full model with the interaction term. All statistical tests were based on two-tailed probability and a significance level set at alpha (α) less than 0.05. Statistical analyses were performed in R v.3.6.1. 

## 3. Results

Selected characteristics of the 3387 study participants on taxane regimens stratified by presence of CIPN are shown in Table 1. Paclitaxel was more frequently used than docetaxel (75.6% vs. 24.4%) among our study population. The mean treatment dose of docetaxel was 70.9 mg/m^2^ (standard deviation (SD) = 11.7), and the mean treatment dose of paclitaxel was 85.5 mg/m^2^ (SD = 51.3). Among all study participants, 54.9% of patients received taxane treatment in combination with a platinum agent and 16.8% in combination with a non-platinum agent. A greater proportion of patients received a platinum agent (70.2%) than non-platinum agent (23.8%) prior to taxane treatment. Approximately a quarter (23.6%) of participants received radiotherapy prior to taxane treatment. A similar proportion (21.2%) received radiotherapy concurrently with taxane treatment. Breast cancer (33.4%) was the most common cancer type among study participants, followed by head and neck cancer (21.7%). Approximately half of the participants ever smoked (45.0%), and 16.5% of patients had a history of diabetes. 

Compared to participants who did not experience CIPN, participants who experienced CIPN were, on average, younger (mean 58.5 vs. 59.5; *p* = 0.043) and had higher BMIs (mean 29.2 vs. 27.9; *p* < 0.001). In addition, greater proportions of participants with CIPN were female (68.5% vs. 55.0%; *p* < 0.001) and self-identified as Black (12.3% vs. 9.8%). Patients with CIPN were more likely to have a history of diabetes (19.8% vs. 14.8%; *p* < 0.001) and less likely to have ever smoked (41.8% vs. 46.7%; *p* < 0.001). Among cancer types, breast cancer was more heavily represented in the group of participants with CIPN (40.4% vs. 30.0%) but a higher proportion of participants without CIPN had head and neck (25.4% vs. 14.0%) or lung and other respiratory (19.4% vs. 15.6%) cancers. Participants with CIPN were more likely to have stage I cancer (13.3% vs. 10.3%) and less likely to have stage IV cancer (17.5% vs. 24.8%).

There was a non-significant difference in the type of taxane treatment received by participants with and without CIPN. Among paclitaxel recipients, those with CIPN received higher average doses of paclitaxel compared to those who did not experience CIPN (mean 94.2 vs. 81.1 mg/m^2^; *p* < 0.001). In contrast, docetaxel recipients without CIPN had higher average doses than those with CIPN (mean 71.4 vs. 69.6 mg/m^2^; *p* = 0.038). Interestingly, patients treated with prior (65.5% vs. 72.5%) or concurrent (49.5% vs. 57.5%) platinum-based chemotherapy regimens had a lower proportion of participants with CIPN. Likewise, there were lower proportions of participants with CIPN who had concurrent (13.5% vs. 25.0%) or prior (18.0% vs. 26.3%) radiation therapy. 

In univariate analyses (Table 2; OR1), Black patients were at higher odds of experiencing CIPN compared to White patients (OR = 1.28; 95% CI = 1.02–1.61), as were female patients (OR = 1.78; 95% CI = 1.53 to 2.07). Compared to patients with normal weight, patients who were overweight or obese were at higher risk for CIPN with ORs of 1.29 (95% CI = 1.07 to 1.55) and 1.66 (95% CI = 1.38 to 1.99), respectively. Other factors associated with a high risk of CIPN included history of diabetes (OR = 1.48; 95% CI = 1.22 to 1.78), higher mean taxane dose (OR = 1.05 per 10 mg/m^2^; 95% CI = 1.03 to 1.06), and higher number of taxane treatment cycles (OR = 1.10; 95% CI = 1.08 to 1.11). Patients who were aged ≥75 years had lower odds of experiencing CIPN (OR = 0.69; 95% CI = 0.53–0.91) compared to patients aged <55 years. Other factors inversely associated with CIPN included ever smoking history (OR = 0.77; 95% CI = 0.66 to 0.89), concurrent platinum therapy compared to no concurrent therapy (OR = 0.67; 95% CI = 0.57 to 0.79), concurrent non-platinum therapy (OR = 0.81; 95% CI = 0.65 to 1.01), and concurrent radiotherapy (OR = 0.47; 95% CI = 0.39 to 0.57). Patients treated with co-chemotherapy regimens had varying mean taxane doses and cumulative taxane doses (Table S2). Notably, after adjustments for age and sex (Table 2; OR2), the associations for Black patients, history of ever smoking, and older age were no longer significant. 

In the final multivariate model (Table 2; OR4), patients at higher odds of experiencing CIPN included those who were female (OR = 1.28; 95% CI = 1.01 to 1.62) and those who were overweight (OR = 1.31; 95% CI = 1.06 to 1.61) or obese (OR = 1.49; 95% CI = 1.21 to 1.83). Other characteristics that remained significant were diabetes history (OR = 1.66; 95% CI = 1.34 to 2.06), higher mean taxane dose (per 10 mg/m^2^) (OR = 1.05; 95% CI = 1.03 to 1.08), higher number of taxane treatment cycles (OR = 1.12; 95% CI = 1.10 to 1.14), concurrent platinum therapy (OR = 0.74; 95% CI = 0.58 to 0.94), and non-platinum therapy (OR = 0.64; 95% CI = 0.49 to 0.83).

When stratifying by paclitaxel vs. docetaxel therapy (Table 3), we observed that the association between BMI and CIPN was stronger among docetaxel recipients (OR = 1.79; 95% CI = 1.15 to 2.78, for overweight and OR = 1.77; 95% CI = 1.14 to 2.74 for obese patients) than paclitaxel recipients (OR = 1.43, 95% CI = 1.13 to 1.81 for overweight and OR = 1.43; 95% CI = 1.13 to 1.81 for obese patients). However, the interaction test was only significant between taxane type and diabetes (*p* = 0.019). The odds ratio for CIPN in patients with a history of diabetes who received docetaxel (OR = 2.63; 95% CI = 1.63 to 4.22) was greater than that for patients with history of diabetes who received paclitaxel (OR = 1.50; 95% CI = 1.17 to 1.92). 

We also examined the associations between CIPN with BMI and history of diabetes stratified by sex (Table 3)**.** In female patients, overweight and obese BMIs were significantly associated with CIPN with ORs of 1.39 (95% CI = 1.07 to 1.81) and 1.59 (95% = 1.23 to 2.06), respectively. No significant association was observed in male patients. Likewise, female patients with a history of diabetes had greater odds of CIPN (OR = 1.81; 95% CI = 1.34 to 2.44) than male patients with a history of diabetes (OR = 1.49; 95% CI = 1.07 to 2.08) when compared to their counterparts without a history of diabetes. However, there was no significant multiplicative interaction between sex and BMI (*p* = 0.931) or history of diabetes (*p* = 0.269). 

Table 4 shows a joint association between categorical BMI and history of diabetes. Among patients with no history of diabetes, overweight and obese patients had higher odds of CIPN than normal-weight patients with ORs of 1.34 (95% CI = 1.07 to 1.68) and 1.68 (95% CI = 1.34 to 2.09), respectively. Normal-weight patients with a history of diabetes were at higher odds of CIPN than normal-weight patients without a history of diabetes (OR = 2.65; 95% CI = 1.62 to 4.35). Likewise, overweight and obese patients with a history of diabetes had higher odds of CIPN compared to normal-weight patients without a history of diabetes with ORs of 2.41 (95% CI = 1.65 to 3.52) and 2.15 (95% CI = 1.59 to 2.91), respectively. Notably, these ORs were not higher when compared to normal-weight patients with a history of diabetes across BMI strata. In other words, when comparing the OR of healthy weight individuals with the OR of overweight (2.41/2.65 = 0.91) or obese patients (2.15/2.65 = 0.81), high BMI was not associated with higher CIPN risk among patients with a history of diabetes. Tests for multiplicative interaction between BMI and history of diabetes were significant (*p* = 0.039).

## 4. Discussion

### 4.1. Demographic Correlates

We found several factors associated with increased prevalence of CIPN in univariate analysis among all patients within the cohort, such as cancer type and cancer stage at first taxane treatment. Among patients with CIPN, breast as well as head and neck cancers were the predominant cancer types. Additionally, patients with stage I cancer were more likely to have CIPN compared to patients with stage IV disease. This is likely the result of a combination of factors including cancer screening to detect early-stage breast cancers, early symptom development with head and neck cancers, and aggressive treatment with standard dose intensity for curative intent in all cancer types. We also found that 50% of patients with female genital and reproductive tract (i.e., gynecologic) cancers developed CIPN while only 30% of patients with non-gynecologic cancers developed CIPN. This is likely related to the high dose of paclitaxel used in adjuvant (175 mg/m^2^) and maintenance (135 mg/m^2^) therapy for ovarian cancers [20,21]. It is worth noting that none of these associations retained statistical significance in the multivariate analysis. 

Our univariate analysis also showed a modest increase in the prevalence of CIPN based on patient demographic groups including Black patients compared with White patients and in female patients compared to their male counterparts. Previous studies have reported conflicting results regarding race as a risk factor for CIPN [22,23]. In our study, Black race was no longer a risk factor for CIPN after adjusting for age and sex. Of note, female sex remained significantly associated with higher risk of CIPN in the multivariate analysis, albeit an OR4 drop to 1.28 from the unadjusted OR1 of 1.78. The underlying mechanism for this association remains unknown. Treatment characteristics, including mean taxane dose and number of cycles, had a positive association with the development of CIPN, as reported elsewhere [7].

### 4.2. Treatment Regimen Correlates 

Unexpectedly, we found that taxane treatment concurrent with chemotherapy or radiotherapy was associated with a lower risk of CIPN and the associations persisted even after adjustment for dose and cycle of taxane treatment as well as several other clinical factors. Platinum agents have been previously reported to be associated with increased risk of CIPN [24]. However, patients treated with co-chemotherapy consisting of a taxane and platinum agent versus taxane alone had a lower risk developing CIPN despite having similar mean taxane dose and cumulative taxane dose. Differences in dosing schedules, treatment adherence, and medication contradictions, as well as clinical and patient characteristics (e.g., physical fitness) uncaptured by our study may have contributed to these correlative associations. Additional research, particularly in clinical-trial settings, is needed to draw a conclusion. 

### 4.3. Correlates among Diabetic and Obese Patients 

Our findings agree with several previous studies in supporting diabetes as an important risk factor for taxane-induced CIPN [2,13]. Adjusting for all other variables, including age, sex, race, BMI, lifetime smoking incidence, mean taxane dose, number of taxane cycles, concurrent chemotherapy, concurrent radiotherapy, or prior chemotherapy, we found that taxane-treated patients with a history of diabetes were more than 1.6 times as likely to develop CIPN when compared to non-diabetic patients. This association was consistently observed across BMI categories. Obesity was an independent risk factor for developing CIPN only among non-diabetic patients. Furthermore, the association of diabetes and taxane-induced CIPN was stronger among docetaxel than among paclitaxel treated patients. 

Separating the effect of BMI from a history of diabetes is paramount due to the high correlation between these two factors. BMI is an established risk factor for developing type 2 DM (T2DM) [25], and the prevalence of adults with diabetes increases from 8% for normal-weight individuals to 43% for individuals with a BMI > 40 [26]. Of note, one quarter of patients with DM will develop diabetic sensorimotor polyneuropathy (DPN). As a result, patients with DM or pre-existing DPN of any grade are often excluded from clinical trials containing CIPN-inducing therapies to avoid its confounding effect [2,27]. Due to these complications, the association between diabetes and CIPN warrants further evaluation. In our study, we found that BMI was associated with CIPN only among non-diabetic patients, while diabetes was associated with CIPN across all BMI categories. Interestingly, our data showed that compared to normal-weight non-diabetic patients, normal-weight diabetic patients had a higher OR of CIPN than overweight or obese diabetic patients. Unfortunately, information on DPN was not available, which precluded us from separating DPN from CIPN. It is possible that some of the patients in our cohort with diabetes may have had DPN prior to receiving taxane. Furthermore, it is unclear whether adherence to pharmacologic diabetes management may have impacted CIPN. We were also unable to accurately classify types of DM in our study (e.g., type 1 DM from type 2 DM, insulin dependent vs. non-insulin dependent). Avoiding taxane regimens can lead to inferior progression-free survival and overall survival in specific patient populations [28], therefore there is a critical need to delineate further associations between DPN and CIPN while exploring prevention strategies for patients at high risk for CIPN. Future studies evaluating CIPN in DM patients should account for type of diabetes, time since diagnosis, type of diabetes management, medication adherence, HbA1c%, and duration of treatment, as these associations may provide clinical guidance and influence therapeutic decisions. 

### 4.4. Strengths and Limitations 

The strengths of our study include the large sample size, inclusion of multiple cancer types and all stages of disease, and consideration of multiple treatment modalities including concurrent combination chemotherapy and concurrent radiotherapy. 

Our study had several limitations. First, our study was based on data from electric health records that were not specifically designed for research. Information misclassification, such as un-documented or mis-reported smoking behavior and comorbidities, may lead to biased estimation of true associations. Other uncaptured confounders, as mentioned above, may also result in false associations. As discussed, our cohort was limited by an inability to classify type of diabetes. However, given the high prevalence of obesity and diabetes in our geographic region, type 2 DM was suggested to account for 90–95% of diabetics in our patient population [25,29]. Absence of other DM-specific variables, including presence of DPN, time since diabetes diagnosis, glycemic control, medication adherence, HbA1c%, and duration of treatment are also limitations of our study. Additionally, CIPN was identified from EHR using a machine-learning model, which likely introduced some misclassifications. However, our model was validated by manual review and 32% of patients in our assessed cohort developed CIPN, which aligns with previous studies reporting 27% of patients reporting CIPN [2]. Lastly, we focused primarily on the first taxane exposure and did not include patients who may have received prior taxane chemotherapy. 

## 5. Conclusions

We found that obesity, a history of diabetes, and female sex were significantly correlated with taxane-induced CIPN. Patients with both cancer and diabetes had more than a two-fold increased risk of developing CIPN after receiving taxane treatment compared to non-diabetic, normal-weight cancer patients. BMI was also associated with increased risk for CIPN among non-diabetic cancer patients. Race was not an independent risk factor for CIPN. Our study findings provide important information for clinical management of patients with cancer during and after taxane treatment. Studies focusing on investigating underlying biological mechanisms for obesity, diabetes, and sex associations are needed to develop preventive and therapeutic strategies to mitigate taxane-induced CIPN. 

## Figures and Tables

**Table 1:** Prevalence of neuropathy by patient demographic and clinical characteristics.

	All Participants (*n* = 3387)	No Neuropathy (*n* = 2283)	Neuropathy Cases (*n* = 1104)	*p*-Value ^a^
Age (years) at first treatment, mean ± SD	59.1 (12.6)	59.5 (12.7)	58.5 (12.4)	0.0428
Age categorical, *n* (%)				0.0497
<55	1141 (33.7)	750 (32.9)	391 (35.4)	
55 to <65	1019 (30.1)	678 (29.7)	341 (30.9)	
65 to <75	877 (25.9)	598 (26.2)	279 (25.3)	
≥75	350 (10.3)	257 (11.3)	93 (8.4)	
Sex, *n* (%)				7.51 × 10^−14^
Male	1375 (40.6)	1027 (45.0)	348 (31.5)	
Female	2012 (59.4)	1256 (55.0)	756 (68.5)	
Race, *n* (%)				0.0692
White	2927 (86.4)	1988 (87.1)	939 (85.1)	
Black	360 (10.6)	224 (9.8)	136 (12.3)	
Other	100 (3.0)	71 (3.1)	29 (2.6)	
BMI, Mean ± SD	28.3 (6.7)	27.9 (6.6)	29.2 (6.8)	2.89 × 10^−8^
BMI categorical, *n* (%)				4.41 × 10^−7^
<18.5	102 (3.0)	75 (3.3)	27 (2.4)	
18.5 to <25	1056 (31.2)	769 (33.7)	287 (26.0)	
25 to <30	1083 (32.0)	731 (32.0)	352 (31.9)	
≥30	1146 (33.8)	708 (31.0)	438 (39.7)	
History of diabetes, *n* (%)				4.28 × 10^−5^
No	2827 (83.5)	1947 (85.3)	880 (79.7)	
Yes	560 (16.5)	336 (14.7)	224 (20.3)	
Ever smoke, *n* (%)				0.00049
No	1650 (48.7)	1060 (46.4)	590 (53.4)	
Yes	1524 (45.0)	1068 (46.8)	456 (41.3)	
Cancer type, *n* (%) ^b^				1.80 × 10^−20^
Breast	1131 (33.4)	685 (30.0)	446 (40.4)	
Head and neck	736 (21.7)	581 (25.4)	155 (14.0)	
Lung and other respiratory	614 (18.1)	442 (19.4)	172 (15.6)	
Gynecological	210 (6.2)	104 (4.6)	106 (9.6)	
Gastrointestinal	177 (5.2)	125 (5.5)	52 (4.7)	
Unknown	127 (3.7)	87 (3.8)	40 (3.6)	
Cancer stage, *n* (%) ^c^				4.75 × 10^−7^
I	384 (11.3)	234 (10.2)	150 (13.6)	
II	455 (13.4)	313 (13.7)	142 (12.9)	
III	461 (13.6)	291 (12.7)	170 (15.4)	
IV	755 (22.3)	569 (24.9)	186 (16.8)	
Unknown	1332 (39.3)	876 (38.4)	456 (41.3)	
Taxane type, *n* (%)				0.0793
Docetaxel	827 (24.4)	578 (25.3)	249 (22.6)	
Paclitaxel	2560 (75.6)	1705 (74.7)	855 (77.4)	
Mean treatment dose ± SD, mg/m^2^	81.9 (45.4)	78.7 (45.5)	88.7 (44.4)	1.69 × 10^−9^
Docetaxel (mean ± SD, mg/m^2^)	70.9 (11.7)	71.4 (11.5)	69.6 (12.1)	0.0382
Paclitaxel (mean ± SD, mg/m^2^)	85.5 (51.3)	81.1 (52.0)	94.2 (48.7)	9.43 × 10^−10^
Co-chemotherapy, *n* (%)				1.10 × 10^−5^
None, *n* (%)	959 (28.3)	592 (25.9)	367 (33.2)	
Platinum, *n* (%)	1860 (54.9)	1313 (57.5)	547 (49.5)	
Other chemotherapy drugs	568 (16.8)	378 (16.6)	190 (17.2)	
Prior chemotherapy, *n* (%)				2.82 × 10^−6^
None, *n* (%)	204 (6.0)	144 (6.3)	60 (5.4)	
Platinum, *n* (%)	2378 (70.2)	1655 (72.5)	723 (65.5)	
Other chemotherapy drugs	805 (23.8)	484 (21.2)	321 (29.1)	
Co-radiotherapy, *n* (%)				1.98 × 10^−14^
No	2668 (78.8)	1713 (75.0)	955 (86.5)	
Yes	719 (21.2)	570 (25.0)	149 (13.5)	
Prior radiotherapy, *n* (%)				9.80 × 10^−8^
No	2587 (76.4)	1682 (73.7)	905 (82.0)	
Yes	800 (23.6)	601 (26.3)	199 (18.0)	

^a^: *p*-values derived from Pearson’s *χ*^2^ tests for independence for categorical variables and Student’s *t* tests for continuous variables comparing self-reported race; ^b^: Top five cancer types are listed. Cancer types were determined using most recent diagnosis code to start of taxane treatment, Patients without diagnosis codes were grouped into “Unknown;” ^c^: Cancer stage was determined from VUMC NAACCR data, which were only available for patients diagnosed at VUMC.

**Table 2:** Association of neuropathy with patient demographics and clinical characteristics.

	*n*	OR1 (95% CI)	OR2 (95% CI)	OR3 (95% CI)	OR4 (95% CI)
Age					
<55	391/1141	Reference	Reference	Reference	Reference
55 to <65	341/1019	0.96 (0.81 to 1.15)	1.06 (0.88 to 1.27)	1.06 (0.88 to 1.27)	1.02 (0.83 to 1.25)
65 to <75	279/877	0.90 (0.74 to 1.08)	1.00 (0.83 to 1.21)	0.99 (0.82 to 1.20)	0.97 (0.78 to 1.21)
≥75	93/350	0.69 (0.53 to 0.91)	0.81 (0.62 to 1.06)	0.83 (0.63 to 1.09)	0.74 (0.54 to 1.01)
Sex					
Male	348/1375	Reference	Reference	Reference	Reference
Female	756/2012	1.78 (1.53 to 2.07)	1.76 (1.51 to 2.05)	1.72 (1.47 to 2.01)	1.28 (1.01 to 1.62)
Race					
White	939/2927	Reference	Reference	Reference	Reference
Black	136/360	1.28 (1.02 to 1.61)	1.18 (0.94 to 1.49)	1.14 (0.90 to 1.43)	1.02 (0.80 to 1.32)
Other	29/100	0.86 (0.56 to 1.34)	0.84 (0.54 to 1.30)	0.88 (0.56 to 1.38)	0.97 (0.60 to 1.56)
BMI					
<18.5	27/102	0.96 (0.61 to 1.53)	0.97 (0.61 to 1.54)	0.97 (0.61 to 1.54)	1.04 (0.61 to 1.77)
18.5 to <25	287/1056	Reference	Reference	Reference	Reference
25 to <30	352/1083	1.29 (1.07 to 1.55)	1.32 (1.09 to 1.59)	1.32 (1.09 to 1.59)	1.31 (1.06 to 1.61)
≥30	438/1146	1.66 (1.38 to 1.99)	1.58 (1.32 to 1.90)	1.58 (1.32 to 1.90)	1.49 (1.21 to 1.83)
History of diabetes					
No	880/2827	Reference	Reference	Reference	Reference
Yes	224/560	1.48 (1.22 to 1.78)	1.67 (1.38 to 2.03)	1.54 (1.26 to 1.88)	1.66 (1.34 to 2.06)
Ever smoke					
No	590/1650	Reference	Reference	Reference	Reference
Yes	456/1524	0.77 (0.66 to 0.89)	0.90 (0.77 to 1.05)	0.92 (0.79 to 1.08)	0.96 (0.80 to 1.15)
Taxane type					
Docetaxel	249/827	Reference	Reference	Reference	Reference
Paclitaxel	855/2560	1.16 (0.98 to 1.38)	1.26 (1.06 to 1.50)	1.26 (1.06 to 1.50)	0.80 (0.62 to 1.01)
Mean taxane dose (per 10 mg/m^2^)		1.05 (1.03 to 1.06)	1.04 (1.02 to 1.06)	1.04 (1.03 to 1.06)	1.05 (1.03 to 1.08)
Number of taxane cycles		1.10 (1.08 to 1.11)	1.09 (1.08 to 1.11)	1.09 (1.08 to 1.11)	1.12 (1.10 to 1.14)
Concurrent chemotherapy					
None	367/959	Reference	Reference	Reference	Reference
Platinum	547/1860	0.67 (0.57 to 0.79)	0.73 (0.61 to 0.86)	0.72 (0.61 to 0.86)	0.74 (0.58 to 0.94)
Non-platinum	190/568	0.81 (0.65 to 1.01)	0.72 (0.57 to 0.89)	0.71 (0.57 to 0.89)	0.64 (0.49 to 0.83)
Concurrent radiotherapy					
No	955/2668	Reference	Reference	Reference	Reference
Yes	149/719	0.47 (0.39 to 0.57)	0.54 (0.44 to 0.66)	0.54 (0.44 to 0.67)	0.77 (0.59 to 1.00)

*n*: counts of patients in category with neuropathy/total number of patients in category; OR1: unadjusted; OR2: adjusted for age and sex; OR3: adjusted for age, sex, and BMI; OR4: adjusted for all variables in table and prior chemotherapy, prior radiotherapy, cancer type, and cancer stage.

**Table 3:** Association of neuropathy with patient demographic and clinical characteristics stratified by type of taxane and sex.

	Paclitaxel Recipients (*n* = 2481)		Docetaxel Recipients (*n* = 804)		Male (*n* = 1329)		Female (*n* = 1956)	
	*n*	OR (95% CI)	*n*	OR (95% CI)	*n*	OR (95% CI)	*n*	OR (95% CI)
BMI								
18.5 to <25	230/794	Reference	57/262	Reference	104/450	Reference	183/606	Reference
25 to <30	266/827	1.19 (0.94 to 1.51)	86/256	1.79 (1.15 to 2.78)	122/488	1.17 (0.83 to 1.64)	230/595	1.39 (1.07 to 1.81)
≥30	337/860	1.43 (1.13 to 1.81)	101/286	1.77 (1.14 to 2.74)	115/391	1.28 (0.89 to 1.83)	323/755	1.59 (1.23 to 2.06)
	*p*-value for interaction = 0.702				*p*-value for interaction = 0.931			
Diabetes								
No	666/2032	Reference	191/699	Reference	252/1041	Reference	605/1690	Reference
Yes	167/449	1.50 (1.17 to 1.92)	53/105	2.63 (1.63 to 4.22)	89/288	1.49 (1.07 to 2.08)	131/266	1.81 (1.34 to 2.44)
	*p*-value for interaction = 0.019				*p*-value for interaction = 0.269			

*n*: counts of patients in category with neuropathy/total number of patients in category; OR: adjusted for all variables in table and prior chemotherapy, prior radiotherapy, cancer type, and cancer stage; *p*-value for interaction derived from calculating the *p*-value of the F-test comparing the reduced model without interaction term and the full model with the interaction term.

**Table 4:** Interaction between BMI and history of diabetes.

	No History of Diabetes (*n* = 2731)		History of Diabetes (*n* = 554)	
	*n*	OR (95% CI)	*n*	OR (95% CI)
BMI				
18.5 to <25	250/962	Reference	37/94	2.65 (1.62 to 4.35)
25 to <30	287/916	1.34 (1.07 to 1.68)	65/167	2.41 (1.65 to 3.52)
≥30	320/853	1.68 (1.34 to 2.09)	118/293	2.15 (1.59 to 2.91)
*p*-value for interaction = 0.039				

*n*: counts of patients in category with neuropathy/total number of patients in category; OR: adjusted for age, sex, race, ever smoking status, mean taxane dose, number of taxane cycles, co-chemotherapy, prior chemotherapy, co-radiotherapy, prior radiotherapy, cancer type, and cancer stage; *p*-value for interaction derived from calculating the *p*-value of the F-test comparing the reduced model without interaction term and the full model with the interaction term.

**Figure A1:**
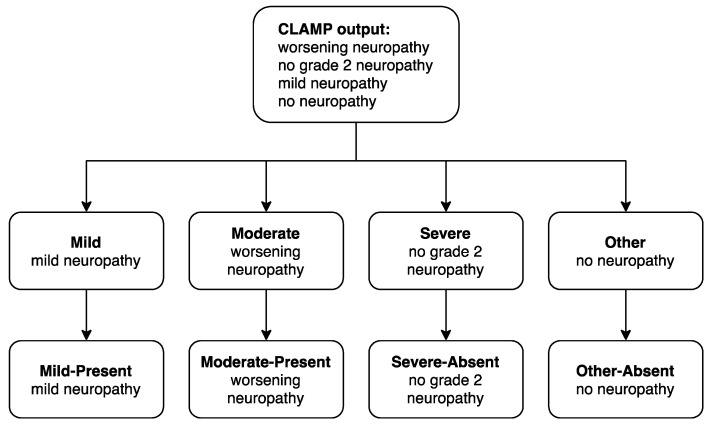
Flowchart showing the categorization of example neuropathy terms extracted by CLAMP from clinical notes.

**Figure A2:**
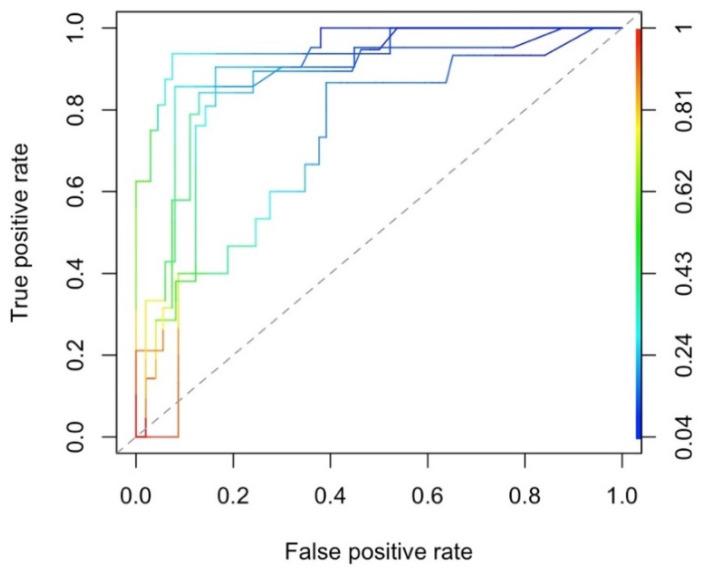
Receiver operating curve of all five folds from the five-fold cross validation of the neuropathy case-control prediction model.

## Data Availability

The data presented in this study are available on request from the corresponding author conditioning on compliance with the VUMC data sharing policy.

## Supplementary Materials

The following supporting information can be downloaded at: https://www.mdpi.com/2072-6694/15/3/754/s1, Table S1: List of chemotherapy agents evaluated.; Table S2: Mean and cumulative taxane dose when administered with co-chemotherapy.

## Appendix A

### Appendix A.1. Machine-Learning Model to Identify Chemotherapy-Induced Peripheral Neuropathy from Electronic Health Records

We identified 3577 individuals who had undergone taxane treatments from electronic health records (EHRs) in Vanderbilt University Medical Center’s Synthetic Derivative. Information about each individuals’ taxane treatment was retrieved from the EHRs, such as type of taxane, length of treatment period, number of treatment cycles, dosage at each administration, and date of administration. A clinician reviewed a subset of 242 patients and identified grade 2 chemotherapy-induced peripheral neuropathy (CIPN).

Terms related to neuropathy were extracted from the individual’s clinical notes during their taxane treatment period with the Clinical Language Annotation, Modeling, and Processing (CLAMP) toolkit [30]. CLAMP outputs a flag that indicates whether the extracted term was negated and will, where possible, include other modifiers in the extracted term, such as severity (e.g., mild neuropathy) or location (e.g., neuropathy in hands). We grouped the extracted neuropathy terms into four severity categories based on the term modifiers with case-insensitive regular expressions, as shown in Table A1. As our goal was to identify grade 2 and above CIPN, we grouped grade 2 and grade 3 CIPN in the “Severe” category.

**Table A1:** Classifying neuropathy terms into severity categories with term modifiers.

	Mild	Moderate	Severe	Other
Modifiers	Mild Grade 1 Grade I Minimal Minor	Moderate Grade 1–2 Grade 1–2 Worsening Progressing Increasing Persistent Ongoing Leg ^a^	Severe Grade 2 Grade 2 Grade 3 Grade 3 Significant Painful	All terms not classified as “mild”, “moderate”, or “severe”

^a^ Neuropathy terms modifiers can include location, e.g., ‘neuropathy in leg’.

We additionally separated the neuropathy terms into ‘present’ and ‘absent’ based on the CLAMP negation flag, giving eight term categories: mild-present, moderate-present, severe-present, other-present, mild-absent, moderate-absent, severe-absent, other-absent. For instance, a term such as ‘worsening neuropathy’ would be classified as moderate-present whereas a term such as ‘no grade 2 neuropathy’ would be classified as severe-absent (Figure A1).

### Appendix A.2. Neuropathy Case-Control Prediction Model

We trained an L1-regularized logistic regression model to predict neuropathy case-control status using the subset of 242 individuals with clinician-reviewed neuropathy case-control labels. Predictors included presence of ICD for neuropathy, log-counts for the eight neuropathy term categories, and log-counts for each of the number of taxane dosage increases and decreases during the taxane treatment course. For each individual, the log-counts for the eight neuropathy term categories were calculated by taking the log of the cumulative counts for each neuropathy term’s category across the individual’s whole EHR. Log-counts of taxane dosage increases and decreases were calculated by taking the log of the cumulative counts of 5% increases and decreases in taxane dosage during the taxane treatment period. We evaluated the area under the receiver operating characteristic curve (AUC) with five-fold cross validation. Statistical analyses were performed in R v.3.4.3.

### Appendix A.3. Performance of Neuropathy Case-Control Prediction Model

The five-fold cross-validation of the neuropathy case-control prediction model had a mean AUC of 0.861 (range: 0.715 to 0.962). Figure A2 shows the receiving operating curves for all five cross-validation folds.
