# Supplementary material for: Correlates of Taxane-Induced Neuropathy, an Electronic Health Record Based Observational Study
Source: Cancers (Basel). 2023 Jan 26;15(3):754. doi: 10.3390/cancers15030754 (PMC9952888; doi:10.3390/cancers15030754)
Supplement: Supplementary file 1 [file cancers-15-00754-s001.zip › cancers-2135375-supplementary.pdf]

Supplementary Materials

Supplemental Table S1. List of chemotherapy agents evaluated.

**Alkylating Agents**	Cyclophosphamide
**Anthracycline**	Doxorubicin, Epirubicin
**Antimetabolite**	5-FU, Cladribine, Capecitabine, Gemcitabine, Cytarabine
**Antimicrotubule agent**	Vinorelbine, Vinblastine, Vincristine, Eribulin mesylate, Ixabepilone
**CDK4/6 Inhibitor**	Palbociclib, Abemaciclib, Ribociclib
**Monoclonal antibody**	Trastuzumab, Pertuzumab, Bevacizumab
**Platinum**	Cisplatin, Carboplatin, Oxaliplatin
**Taxane**	Paclitaxel, Docetaxel, Nab-paclitaxel / albumin-bound paclitaxel, Liposomal pacilitaxel
**Topoisomerase inhibitor**	Etoposide, Teniposide, Irinotecan, Topotecan, Camptothecin
**Tyrosine Kinase Inhibitor**	-tinib

Supplemental Table S2. Mean and cumulative taxane dose when administered with co-chemotherapy.

**Co-Chemotherapy**	**N**	**Mean Taxane Dose Per Treatment Cycle (mg/m2)**	**Cumulative Taxane Dose (mg/m2)**
None	660	82.16322	525.9764
Alkylating Agents	289	77.28803	441.4465
Anthracyclines	112	83.00521	847.1465
Anti-microtubule Agents	41	75.72213	1014.7650
Antimetabolite agents	264	84.61523	590.1076
CDK inhibitors	6	79.09842	889.0724
Monoclonal antibodies	729	91.00079	624.5227
Platinum agents	2233	82.26618	500.4511
Tyrosine kinase inhibitors	84	100.26901	552.6470

N: counts of patients treated with a specific chemotherapy regimen.
